# A Novel Monoclonal Antibody against PD-1 for the Treatment of Viral Oncogene-Induced Tumors or Other Cancer

**DOI:** 10.3390/cancers16173052

**Published:** 2024-09-01

**Authors:** Xu Xu, Shih-Long Yan, Yi-Te Yo, Peiyu Chiang, Chan-Yen Tsai, Lih-Ling Lin, Albert Qin

**Affiliations:** 1 Research Department, PharmaEssentia Corporation, Taipei 115, Taiwan; 2 Medical Research & Clinical Operations, PharmaEssentia Corporation, Taipei 115, Taiwan

**Keywords:** P1801, PD-1, anti-PD-1 antibody, pharmacokinetics, pharmacodynamics

## Abstract

**Simple Summary:**

Viral infection poses cancer risk and the PD-1/PD-L1 immune check point plays a critical role in this process. Despite the success of the existing anti-PD-1 and PD-L1 antibodies, significant challenges remain due to drug resistance and serious side effects. We have developed a novel anti-PD-1 antibody P1801 which has significant PD-1 blocking and antitumor activities. It displayed unique binding properties distinctive from pembrolizumab and nivolumab with limited ADCC- and CDC-mediated lysis of normal immune cells. We are initiating a Phase 1 clinical study to test its combination use with ropeginterferon alfa-2b, a new-generation interferon-alfa-based therapy which also has antiviral and antitumor activities, for the treatment of cancer.

**Abstract:**

Programmed cell death 1 (PD-1) and programmed death-ligand 1 (PD-L1) interact to form an immune checkpoint fostering viral infection and viral oncogene-induced tumorigenesis. We generated a novel anti-human PD-1, humanized monoclonal antibody P1801 and investigated its pharmacologic, pharmacokinetic (PK), and pharmacodynamic properties. In vitro binding assays revealed that P1801 uniquely binds to human PD-1 and inhibits its interaction with PD-L1/2. It showed a minor effect on the induction of antibody-dependent cell-mediated cytotoxicity (ADCC) and complement-dependent cytotoxicity (CDC). P1801 significantly induced the release of IL-2 from activated T-cells but not from nonactivated T-cells. A dose-dependent linear PK profile was observed for the cynomolgus monkeys treated with repeated doses of P1801 at 5 mg/kg to 200 mg/kg once weekly. A four-week repeat-dose toxicity study revealed that P1801 given weekly was safe and well tolerated at doses ranging from 5 to 200 mg/kg/dose. No pathological abnormalities were noted. In humanized PD-1 mice harboring human PD-L1-expressing colon tumor cells, P1801 administered intraperitoneally twice per week at 12 mg/kg significantly inhibited tumor growth and prolonged mouse survival. P1801 displayed unique binding properties different from pembrolizumab and nivolumab. Therefore, it showed distinctive immunological reactions and significant antitumor activities. We are initiating a Phase 1 clinical study to test its combination use with ropeginterferon alfa-2b, which also has antiviral and antitumor activities, for the treatment of cancer.

## 1. Introduction

Cellular transformation to cancer cells can result from introduction of viral oncogenes, activation of cellular proto-oncogenes and inactivation of tumor suppressor genes among various causes [1]. Although the human body is supposed to have immunosurveillance [2] and the cell cycle-based anticancer surveillance system [3], cancer cells can find ways to escape the surveillance to grow into cancer. The activation of the immune checkpoint via an interaction of programmed cell death 1 (PD-1) and programmed death-ligand 1 (PD-L1) is a common way of cancer cells escaping the immunosurveillance. The interaction triggers signals which lead to the suppression of immunity and helps cancer cells and viruses carrying viral oncogenes escape immune responses against them [4,5,6]. PD-L1 is upregulated in tumor cells, interacting with PD-1 on immune cells to suppress T-cell-mediated immunologic responses [7]. Anti-PD-1 and anti-PD-L1 blocking antibodies can interrupt the PD-1/PD-L1 interaction network to activate the immune responses and elicit therapeutic anticancer effects [6,8]. Several anti-PD-1/PD-L1 antibodies have been used clinically as approved immunotherapies for the treatment of cancer [9,10,11,12]. 

The interaction of viral oncogenes with the PD-1/PD-L1 interaction network is known to accelerate tumorigenesis and cancer growth. For example, the Epstein–Barr virus (EBV) latent membrane protein 1 (LMP1), a critical viral oncoprotein, can foster an immunosuppressive tumor microenvironment (TME) by upregulating PD-L1 and immunosuppressive cytokines in EBV-related classical Hodgkin lymphomas (cHLs) [13]. The TME of EBV-related cHLs contains higher expression levels of PD-L1 than that of EBV-unrelated cHLs. Blocking the PD-1/PD-L1 signaling relieves the immunosuppression. Anti-PD-1 therapeutic antibodies have been shown to have antitumor activities and are approved for the treatment of cHLs. In addition, mutant Kirsten rat sarcoma viral oncogene homologue (KRAS), which is commonly present in non-small-cell lung cancer (NSCLC), upregulates PD-L1 expression in lung adenocarcinoma cells [14]. The KEYNOTE-042 trial showed that the anti-PD-1 antibody pembrolizumab as a first-line treatment improved overall survival (OS) compared to chemotherapy in patients with NSCLC [15]. Anti-PD-1/PD-L1 antibodies have also been assessed for their efficacy in the other viral related carcinomas, such as hepatitis B virus (HBV)-related hepatocellular carcinoma (HCC) [16]. Chronic viral infections often lead to a decreased T-cell-mediated antiviral response, i.e., T-cell exhaustion, and PD-1 blockade can reverse this response [17]. Chronic HBV infection often causes elevated levels of PD-L1 via the PTEN/β-catenin/c-Myc signaling pathway [18], leading to T-cell exhaustion and the promotion of the formation of an immunosuppressive TME [19,20,21]. The TME of HBV-associated HCC was shown to possess more severe immunosuppression and exhaustion than that of non-virus-associated HCC [22], which contributes to the poor prognosis. Blocking PD-1 activity has been shown to prevent HBV infection by reversing exhaustion, enhancing the production of interferon (IFN)-gamma, and inducing the proliferation of peripheral blood mononuclear cells (PBMCs) [23,24]. Anti-PD-1 therapy was also suggested to partially rescue virus-specific, dysfunctional B cells and lead to a decline or sera-clearance of HBV surface antigen (HBsAg) in patients with chronic HBV infection [25,26].

Despite the success and promise of the existing anti-PD-1 and anti-PD-L1 antibodies, challenges resulting from treatment resistance and immune-related side effects remain. The side effects can be serious and include cardiac toxicity, severe cytokine release syndrome, pneumonitis, hepatitis, thyroiditis and endocrine dysfunction [27,28,29,30,31,32,33,34,35,36]. These immune-related side effects are likely due to an expansion of the T-cell repertoire and autoantibody production by B cells [37]. As part of our immunotherapy development programs, we have been putting in efforts to generate an anti-PD-1 antibody that can be used in combination with our other drug candidates for the treatment of cancer and viral hepatitis B. A promising, novel, recombinant anti-human PD-1 IgG4 humanized monoclonal antibody against PD-1, P1801, was derived from library screening and produced in a cell expression system. Here, we report the generation, nonclinical pharmacology, pharmacokinetics (PK), and pharmacodynamics of P1801 and our plan to combine P1801 with ropeginterferon alfa-2b, another antiviral and anticancer agent.

## 2. Materials and Methods

### 2.1. Construction and Generation of P1801

The coding region of the anti-PD-1 humanized antibody P1801 was first selected from murine hybridoma screening in NZBWF1/J female mice immunized with a purified fusion protein, hPD-1-mFc. Antibody humanization and codon optimization were then conducted to remove immunogenic content and improve translation efficiency. The DNA sequence of human PD-1 was modified from the mRNA sequence (NM_005018.3) for the expression of the hPD-1-mFc fusion protein. The expression vectors of P1801 were subsequently cloned and amplified in an *Escherichia coli* system. A CHO cell expression system was used to produce P1801.

### 2.2. BLI-Based P1801 Epitope Binning Assay

Biolayer interferometry (BLI), an optical analytical technique detecting interference patterns in light waves, was employed for this study. The resulting spectral shift, observed at the detector, was documented on a sensorgram as a change in wavelength (nm shift). Real-time monitoring of the interference pattern facilitated the acquisition of kinetic data pertaining to molecular interactions. Murine anti-human PD-1 (PD1RB) purified antibodies were preincubated with PD-1/His antigen (10 μg/mL antibody; 0.336 μg/mL PD-1 antigen) for 1 h. The anti-human IgG (Fc) sensors were loaded with each benchmark antibody at 3 μg/mL. The biosensors underwent a series of steps: baseline (30 s), loading of the benchmark antibody (3 μg/mL, 700 s), and quenching (480 s), followed by another baseline step (300 s). Subsequently, an association phase of blocking Ab (10 μg/mL) plus PD-1 (0.336 μg/mL) occurred over a 600 s period. The Octet BMIA measured the association of the preformed antibodies plus antigen complex and compared it to the association of antigen alone. A higher binding response indicated non-overlapping epitopes, while a lower response suggested epitope overlap.

### 2.3. Direct Binding ELISA for the Assessment of Binding Specificity

Antigens including human PD-L1 (Sino Biological, Beijing, China, 10084-H08H), human PD-L2 (Sino Biological, 10292-H08H), human CTLA-4 (Sino Biological, 11159-H08H), human CD28 (Sino Biological, 11524-HCCH), mouse PD-1 (Sino Biological, 50124-M08H), cynomolgus PD-1 (Sino Biological, 90311-C08H), human PD-1 (Sino Biological, HPLC-10377-H08H), and hPD-1-mIgG Fc (PharmaEssentia) were coated overnight at a predetermined concentration and blocked with 1% BSA for 2 h. P1801 was then incubated with antigen at 25 °C for 1 h. A peroxidase-conjugated goat anti-hIgG Fc secondary antibody (Jackson Lab, 109-035-098) and TMB peroxidase substrate (KPL, 5120-0080) were used for color development.

### 2.4. Measurement of the Inhibition of PD-1/PD-L1 and PD-1/PD-L2 via Competitive Binding ELISA

Antigens including human PD-L1 (Sino Biological, 10084-H08H) and human PD-L2 (Sino Biological, 10292-H08H) were coated overnight at a predetermined concentration and blocked with 1% BSA for 2 h. Biotin-hPD-1-mIgG Fc (PharmaEssentia) was used as a binder at a concentration of 0.025 μg/mL. Serial dilutions of P1801 were added as a competitor at a starting dose of 0.5 μg/mL, after which the cells were inoculated with the antigen and binder at 25 °C for 2 h. An anti-streptavidin HRP secondary antibody (Thermo, 21130) and TMB peroxidase substrate (KPL, 5120-0080) were used for color development.

### 2.5. In Vitro Affinity of P1801 for FcRn, C1q, and Human PD-1

A Series S Sensor Chip CM5 (GE Healthcare, BR-1005-30) was used for the immobilization of FcRn and C1q. FcRn and C1q were immobilized at 25 °C. HBS-EP was used as the running buffer. The sensor chip surfaces of flow cells 1 and 2 were activated by freshly mixing 50 mmol/L N-hydroxysuccinimide and 200 mmol/L 1-ethyl-3-(3-dimethylaminopropyl) carbodiimide hydrochloride for 200 s. FcRn or C1q diluted in 30 mmol/L NaAC (pH 4.5) was injected into flow cell 2 to achieve conjugation of the appropriate response unit. Flow cell 1 was used as a blank. After the amine coupling reaction, the chip surface was blocked with a 200 s injection of 1 mol/L ethanolamine hydrochloride. Affinity measurements of FcRn or C1q were performed at 25 °C. The running buffer was HBS-EP+ (10 mM HEPES, 500 mM NaCl, 3 mM EDTA, 0.05% Tween 20, and pH 6.0). Diluted P1801 was injected over the surface of flow cells 1 and 2 as the association phase, followed by injecting running buffer as the dissociation phase. Affinity measurements of human PD-1 were performed at 25 °C, and the running buffer was HBS-EP (10 mM HEPES, 150 mM NaCl, 3 mM EDTA, 0.05% Tween 20, and pH 7.4). Diluted P1801 (2 µg/mL) was injected over the surface as the capture phase, and human PD-1 proteins were injected over the surface as the association phase, followed by injecting running buffer as the dissociation phase.

### 2.6. In Vitro Antibody-Dependent Cell-Mediated Cytotoxicity (ADCC) Assay

PBMCs isolated from healthy donors were seeded into culture flasks at a density of 1.25 × 10^6^ per ml and then incubated overnight. Target cells, i.e., Raji-hPD-1 cells (InvivoGen, San Diego, California, USA, raji-hpd1), were labeled with carboxyfluorescein succinimidyl ester (Thermo Fisher Waltham, MA, USA, C34554) for 15 min at 37 °C. The cells were then preincubated in the absence (untreated) or presence of the test agent, negative control (Human IgG4 Isotype Control Recombinant Antibody, BioLegend, San Diego, CA, USA, 403702), positive control-1 (0.016 to 10 μg/mL, Human IgG1 against human PD-1, InvivoGen, San Diego, CA, USA, hpd1ni-mab1), or positive control-2 (10 μg/mL, Human IgG1 against human CD20, InvivoGen, hcd20-mab1) for 30 min at 37 °C. PBMCs were added at an effector-to-target-cell ratio of 25:1. After incubating for four hours at 37 °C, the PBMCs and target cells were collected via centrifugation. The supernatant was removed, and 250 μL of propidium iodide solution (Sigma, Shanghai, China, P4170) was added. The mixture was subsequently incubated at room temperature in the dark for 10 min. The samples were then analyzed via flow cytometry.

ADCC activity was represented by the percent ADCC (%ADCC) according to the following formula:

$$\%ADCC=\left(\frac{\%\;live\;cell\;of\;untreated-\%\;live\;cells\;of\;P1801\;treated}{\%\;live\;cell\;of\;untreated}\right)\;\times \;100\%$$

### 2.7. In Vitro Complement-Dependent Cytotoxicity (CDC) Assay

The target cells, i.e., Raji-hPD-1 cells (InvivoGen, raji-hpd1), were preincubated in the absence (untreated) or presence of P1801, a negative control (human IgG4 isotype control antibody, BioLegend^®^, 403702), positive control-1 (0.016 to 10 μg/mL, human IgG1 against human PD-1; InvivoGen, hpd1ni-mab1), or positive control-2 (10 μg/mL, human IgG1 against human CD20; InvivoGen, hcd20-mab1) for 30 min at 37 °C. Then, 5% (*v*/*v*) serum was added. After incubating for two hours at 37 °C, the target cells were collected via centrifugation. The supernatant was removed, and 250 μL of PI solution was added. The mixture was subsequently incubated at room temperature in the dark for 10 min. The samples were then analyzed via flow cytometry. The CDC activity was demonstrated by percent CDC, which is the percentage of the PI positive region.

### 2.8. In Vitro Receptor Occupancy Assay

PBMCs isolated from healthy donors were stimulated to express PD-1 with Dynabeads^®^ Human T-Activator CD3/CD28 (Life Technologies, Carlsbad, CA, USA 11131D). PBMCs containing activated CD3^+^ T-cells were incubated with P1801 (0.01, 0.1, or 1 μg/mL; PharmaEssentia, Taiwan, China), nivolumab (0.01, 0.1, or 1 μg/mL; Bristol-Myers Squibb, New York, NY, USA), or the hIgG4 isotype negative control (1 μg/mL; human IgG4 Isotype Antibody, BioLegend, 403702). An R-phycoerythrin-conjugated mouse anti-human IgG4 Fc antibody (SouthernBiotech, Birmingham, AL, USA, 9200-09) and an allophycocyanin-conjugated mouse anti-human CD3 antibody (BioLegend, 300412) were used as the secondary antibodies for the flow cytometry analysis.

### 2.9. Receptor Occupancy Assay in Cynomolgus Monkeys

A total of 12 cynomolgus monkeys were assigned to receive P1801 intravenous infusion at doses of 1, 5, or 20 mg/kg. Blood was collected for receptor occupancy analysis from all surviving animals at pre-dose, 0.0 (mediately after infusion was complete), 24 h post-dose, and 168, 336, 672, 1008 and 1344 h post-dose. Peripheral blood lymphocyte populations were identified via flow cytometry using a heterogeneous lymphocyte gating strategy consisting of CD45 fluorescence staining and/or side-scatter characteristic (SSC) demarcation (CD45^bright^SSC^low^) to delineate the lymphocyte populations. The following lymphocyte subsets were targeted for analysis via flow cytometry: total T-cells (CD3^+^SSC^low^) and P1801-bound T-cells (anti-P1801^+^CD3^+^SSC^low^). FITC-conjugated anti-human IgG4 Fc was used as the secondary antibody for flow cytometry. The total percentage of P1801 bound to CD3^+^ T-cells was assessed and calculated based on the median fluorescence intensity in the FITC channel.

### 2.10. In Vitro Cytokine Response Assay

For the cytokine response assay in T-cells activated by superantigen, whole-blood cells from healthy donors were preincubated with P1801 (0.04, 0.2, 1, 5, and 25 μg/mL; PharmaEssentia), nivolumab (0.04, 0.2, 1, 5, and 25 μg/mL; Bristol-Myers Squibb) or the hIgG4 isotype negative control (0.04, 0.2, 1, 5, and 25 μg/mL; human IgG4 Isotype Antibody, BioLegend, 403702) for 60 min and subsequently stimulated with staphylococcus enterotoxin type B (SEB; 0.1 μg/mL) for 48 h. The supernatants were then collected for the quantitation of IL-2 using a human IL-2 Quantikine ELISA Kit (R&D Systems, Minneapolis, MN, USA, D2050/S2050).

For the nonactivated T-cell cytokine response assay, human PBMCs from healthy donors were incubated with P1801 (1, 10, or 100 μg/mL) or a positive control (1 μg/mL anti-human CD3 antibody or 1 μg/mL anti-human CD28 antibody) at 37 °C for 24 h. The negative control (NC) was untreated PBMCs. The secretion of cytokines, including IFN-γ, IL-1β, IL-2, IL-4, IL-6, and IL-8, in the supernatant was quantified via a BD™ Cytometric Bead Array (BD Biosciences, 551809, 551810, 559279, and 558277) and flow cytometry. The percentage of released cytokines was calculated with the following formula:

$$Relative\;cytokine\;Release\left(\%\right)=\left(\frac{Final\;resiult\;of\;P1801-Final\;result\;of\;negative\;control}{Final\;resiult\;of\;positive\;control-Final\;result\;of\;negative\;control}\right)\;\times \;100\%$$

### 2.11. PK, Immunogenicity, and Toxicokinetics of P1801 in Cynomolgus Monkeys

The monkey study was conducted in COVANCE-Shanghai, China. All procedures complied with the local animal welfare legislation, the Guide for the Care and Use of Laboratory Animals, and an approved protocol by the local Institutional Animal Care and Use Committee (IACUC, No.: 20-011). The monkeys were housed by sex up to three animals per cage and individually housed for study related procedures. The environment was controlled at a temperature range of 21 to 26 °C, a relative humidity of 45 to 91%, eight or greater air changes per hour, and a 12 h light/12 h dark cycle. Diet was provided two times daily. Water was provided ad libitium.

For the single dose PK study, a total of 12 cynomolgus monkeys, including 6 males and 6 females, were randomized to receive a P1801 (PharmaEssentia) single infusion at doses of 1, 5, or 20 mg/kg (4 monkeys per group). P1801 was administered in a 30 min intravenous infusion. Serum was collected pre-dose (−1 h) and at 0 (right after infusion finished), 2, 8, 24, 48, 72, 168, 240, 336, 504, 672, 840, 1008, and 1344 h post-dose. ELISA was used to measure the serum concentration of P1801. C_max_, T_max_, AUC_0-last_, AUC_0-inf_, CL, V_ss_, and T_1/2_ were obtained via noncompartment analysis using Phoenix WinNonlin (Certara USA, Inc., Radnor, PA, USA). No monkey was sacrificed in the single-dose PK study. All animals were transferred to the storage cavern after the last blood collection.

For the four-week repeat dose immunogenicity and toxicokinetic study, a total of 32 cynomolgus monkeys, including 16 males and 16 females, were randomized to receive vehicle control (PharmaEssentia, identical formulation with P1801 except for the active compound) or P1801 at 5, 50, or 200 mg/kg via intravenous infusion once weekly for 5 doses in total. At the initiation of dosing, the animals were 2.2 to 2.5 years old, and their body weights ranged from 2.0 to 2.3 kg for males and 1.9 to 2.4 kg for females. The study period included a 4-week treatment period and a 4-week post-treatment recovery period. Serum samples for PK assessment at the 1st and 4th dose were collected pre-dose and at 1, 6, 24, 48, and 96 h post-dose. Serum samples for PK assessment at the 2nd and 3rd doses were collected pre-dose and at 6 h post-dose. Serum samples for PK assessment at the 5th dose were collected pre-dose and at 6, 24, 72, 168, 336, 504, and 672 h post-dose. ELISA was used for the measurement of the P1801 concentration. C_max_, T_max_, AUC_0−last_, AUC_0−inf_, CL, V_ss_, and T_1/2_ and the accumulation ratio (calculated as C_max_ or AUC_0−t_ at the 4th dose)/(C_max_ or AUC_0−t_ at the 1st dose) were calculated using Phoenix WinNonlin (Certara USA, Inc.). Serum for anti-drug antibody (ADA) assessment was collected before dosing, on Days 15 and 29 after the 1st dose, and on Days 12 and 26 after the last dose. ADA measurement was performed using the MesoScale Discovery platform (Meso Scale Diagnostics, LLC). Briefly, acid dissolution was used to extract anti-P1801 antibody from the serum. Affinity capture was subsequently used to remove free P1801. Anti-P1801 antibody was eluted via acidification and incubated with a mixture containing biotinylated P1801 and sulfo-tagged P1801 to form a complex of sulfo-tagged P1801-ADA-biotinylated P1801. The complex was conjugated with streptavidin and measured using an MSD Sector^TM^ Imager (Meso Scale Diagnostics, LLC). Adverse effects and clinical abnormalities were monitored throughout the 4-week treatment period and a 4-week post-treatment recovery period. Clinical observations included body weight, food consumption, vital signs, ophthalmic and neurological examinations, and electrocardiograms. Clinical hematology, chemistry, and coagulation tests were performed. The animals were sacrificed for clinical pathology examination on Day 26 after the last dose based on the protocol approved by IACUC (No.: 20-011). On Day 32 of the dosing phase, three animals/sex/group, having been fasted overnight, were anesthetized with Zoletil and xylazine, exsanguinated, and necropsied. On Day 26 of the recovery phase, all surviving animals, having been fasted overnight, were anesthetized with Zoletil and xylazine, exsanguinated, and necropsied. Macroscopic and microscopic examinations were performed under the supervision of a pathologist.

### 2.12. Antitumor Assessment in Mice

The mice study was conducted in Crown Bioscience, Jiangsu, China. All procedures complied with the local animal welfare legislation, the Guide for the Care and Use of Laboratory Animals, and an approved protocol by the local IACUC (No.: AN-1903-05-514). The environment was controlled at a temperature range of 20 to 26 °C, a relative humidity of 40 to 70%, and a 12 h light/12 h dark cycle. Diet and water were provided ad libitium. A total of thirty human PD-1 knock-in HuGEMM mice with a C57BL/6 background (Shanghai Model Organisms Center) were used. The age of the animals at study initiation was 6 to 8 weeks. The mice were preinoculated subcutaneously with HuCell MC38 tumor cells (1 × 10^6^). The administration of the study drug was initiated when the tumors reached a mean volume of approximately 65 mm^3^. Animals were randomized to receive intraperitoneal injection of human IgG4 (hIgG4; Crownbio) at 12 mg/kg, P1801 (PharmaEssentia) at 12 mg/kg, or nivolumab (Bristol-Myers Squibb) at 12 mg/kg twice weekly for a total of 6 doses. The date of randomization was denoted as Day 0. Tumor volume, mobility, food and water consumption, changes in body weight, and any other abnormalities were investigated until 50 days after the 1st dose of the study drug. The mice were euthanized via CO_2_ inhalation based on the approved IACUC protocol when the individual tumor volume exceeded 3000 mm^3^. Tumor weight was measured at the end of the study. Measurements of tumor volume was performed twice weekly in two dimensions using a caliper, and tumor volume, expressed in mm^3^, was calculated using the following formula: V = (L × W × W)/2, where V is the tumor volume, L is the tumor length (the longest tumor dimension) and W is the tumor width (the longest tumor dimension perpendicular to L).

## 3. Results

### 3.1. Distinct Epitope Specificity of P1801 Compared to Pembrolizumab and Nivolumab

Nine lead clones of PD-1RB were selected from a pool of 60 sorted hybridoma clones derived from NZBWF1/J female mice, demonstrating robust binding activity to human PD-1 expressed on CHOS cells in the FACS assay. The final top clone, PD-1RB-M59, was identified through comprehensive evaluations of sequences of the complementarity-determining region (CDR), epitope binning studies, binding affinity assessments, primary ELISA, and competitive ELISA. Subsequently, the cDNA sequences from PD-1RB-M59 were humanized, leading to the generation of the P1801 antibody. To further investigate the epitope specificity, a biomolecular interaction analysis was conducted using BLI. The epitope binning study of purified PD-1RB-M59 anti-PD-1 monoclonal antibody was performed to assess potential cross-interference with benchmark human IgG4 antibodies, B1C1, and 5C4, corresponding to the CDR regions of pembrolizumab and nivolumab, respectively. In comparison to both benchmark antibodies, the lowest degree of overlap was demonstrated by PD1RB-M59, indicating a distinct epitope. Although partial blocking activity was observed (Supplementary Figure S1), the results highlight the unique epitope specificity of P1801 in comparison to pembrolizumab and nivolumab.

### 3.2. Molecular Properties of P1801

**Figure 1:**
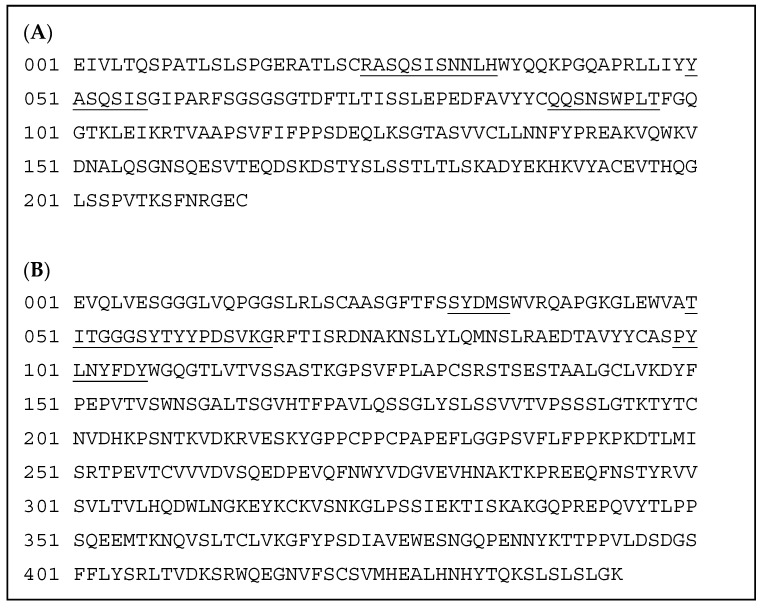
The amino acid sequences of the light chains (**A**) and heavy chains (**B**) of P1801. Underlined text: mouse sequence.

P1801 is an immunoglobulin gamma subclass 4 (IgG4), humanized monoclonal antibody directed against human PD-1. P1801 consists of two kappa light chains with 234 residues and two heavy chains with 464 residues (Figure 1). The CDR1, CDR2, and CDR3 originating from mouse anti-human PD-1 monoclonal antibody were inserted into a human IgG4 framework. The S228P mutation was introduced into the human IgG4 framework to prevent IgG4 arm exchange [38]. One N-linked glycosylation site is present in the heavy chain constant region at position ASN-294. The molecular weight of P1801 is 146,799 Da.

### 3.3. Binding Specificity and the Inhibition of Ligand Binding

**Figure 2:**
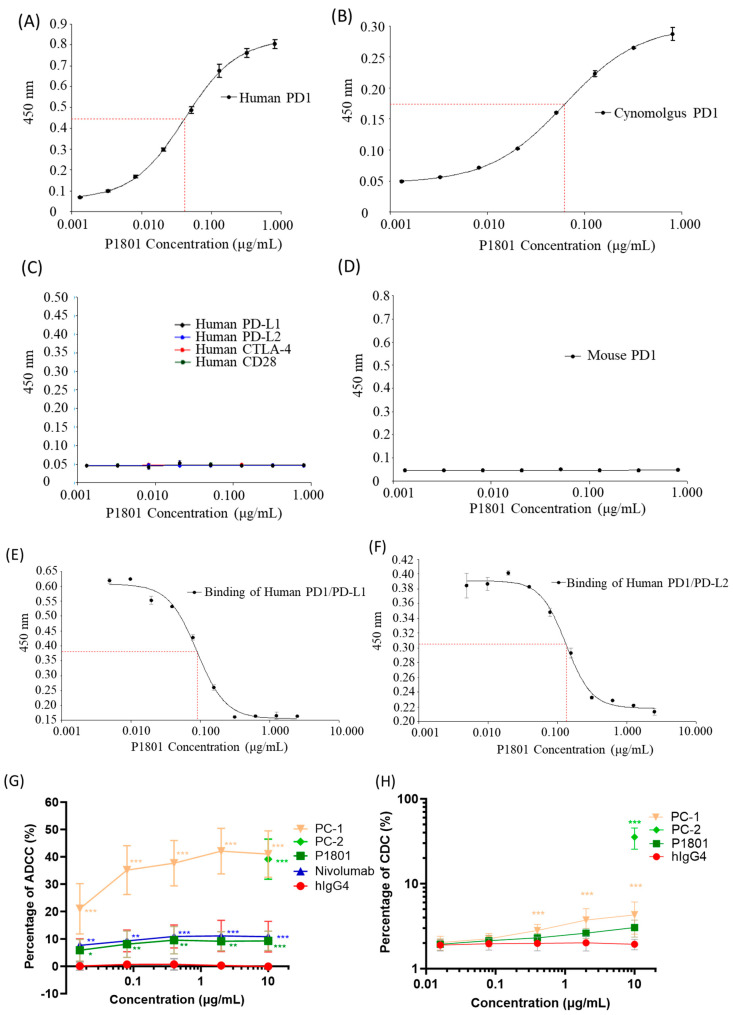
P1801 in vitro binding specificity and absence of cytotoxicity. P1801 bound specifically to human PD-1 (**A**) and cynomolgus PD-1 (**B**) but not to human PD-L1, human PD-L2, human CTLA-4, human CD28 (**C**), or mouse PD-1 (**D**) according to an in vitro ELISA. P1801 significantly inhibited the interaction between PD-1 and PD-L1 (**E**) or PD-L2 (**F**) according to a competitive ELISA. Limited induction of ADCC (**G**) but no effect on CDC activation (**H**) were observed in Raji-hPD-1 cells treated with P1801 in the in vitro cell-based analyses. ADCC: antibody-dependent cell-mediated cytotoxicity; CDC: complement-dependent cytotoxicity. NC: negative control; PC-1: positive control 1; PC-2: positive control 2; * *p* < 0.05, ** *p* < 0.01; and *** *p* < 0.001 compared to the NC. The dotted red line represents the half-maximal effective concentration (EC50) in A and B; or half-maximal inhibitory concentration (IC50) in E and F.

An in vitro binding assay revealed specific binding between P1801 and human PD-1, with a half maximal effective concentration (EC50) of 0.57 nM (Figure 2A), while no interaction was observed between P1801 and human PD-L1, PD-L2, CTLA-4, or CD28 (Figure 2C). Species cross-reactivity was also evaluated. Due to the high conservation between human PD-1 and cynomolgus PD-1 (GenBank NP_001271065.1, 96% identity in the extracellular domain), P1801 was shown to cross-react with cynomolgus PD-1, with an EC50 of 0.44 nM (Figure 2B). No cross-reactivity was observed between P1801 and mouse PD-1 (Figure 2D). An in vitro competitive binding assay revealed the capacity of P1801 to inhibit the interaction between human PD-1 and PD-L1 or PD-L2, with half maximal inhibitory concentrations (IC50s) of 0.57 nM and 0.90 nM, respectively (Figure 2E,F). Similar to pembrolizumab, P1801 interacts weakly with FcRn and C1q [39]. The Kd values for P1801 binding to FcRn or C1q were 253 nM and 56.8 nM, respectively, which were much greater than that for P1801 binding to PD-1 (2.5 nM, Supplementary Table S1). Weak interactions with C1q suggested the limited ability of P1801 to induce CDC. An in vitro assay confirmed a low level of ADCC (Figure 2G) and no effect on the induction of CDC when PBMCs were treated with P1801 (Figure 2H). The strength of the difference in ADCC activity induced by P1801 was comparable to that induced by nivolumab. The representative results of flow cytometry in ADCC and CDC analysis are presented in Supplemental Figures S2 and S3. 

### 3.4. Tissue Cross-Reactivity of P1801 in Humans and Cynomolgus Monkeys

The tissue cross-reactivity of P1801 was assessed via immunohistochemistry (IHC) on forty tissue panels from human and cynomolgus monkeys. Specific positive membranous staining for P1801 was observed on lymphocytes but not on other cell types (Supplementary Table S2). Lymphocytes, which were positively stained in both humans and cynomolgus monkeys, were predominantly found in lymphoid tissues and/or tissues with associated lymphoid structures. A degree of concordance was present in the positive staining observed in the human and cynomolgus monkey tissues and/or tissue structures. The intensity of the observed staining and tissue distribution was generally lower in the cynomolgus monkeys.

### 3.5. In Vitro and In Vivo Pharmacodynamic Activity

**Figure 3:**
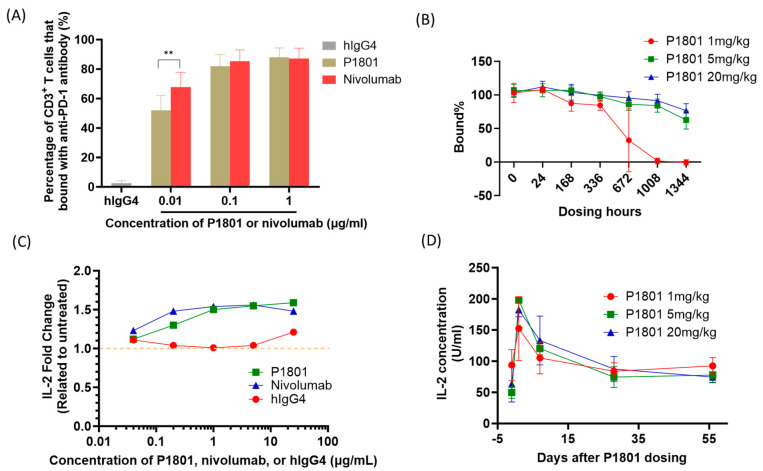
P1801 PD-1 receptor occupancy profile and induction of IL-2 release. A dose-dependent PD-1 receptor occupancy profile was observed for human activated CD3^+^ T-cells treated with P1801 (**A**) and for CD3^+^ T-cells isolated from cynomolgus monkeys treated with P1801 (**B**). IL-2 production in SEB-stimulated human whole-blood cells was enhanced by P1801 and nivolumab in a dose-dependent manner in an in vitro cell-based assay (**C**). According to the ex vivo results, the mean IL-2 level in SEB-induced white blood cells from P1801-treated cynomolgus monkeys was greater after treatment with P1801 than before treatment. White blood cells were separated from monkeys and treated with SEB for three days, after which IL-2 was determined via ELISA (**D**). ** *p* < 0.01.

Both in vitro and in vivo receptor occupancy assays revealed dose-dependent binding of P1801 to PD-1, which is expressed on activated CD3^+^ T-cells. P1801 was found to occupy more than 80% of the T-cells that expressed PD-1 in an in vitro receptor occupancy assessment when cells were treated with P1801 at 0.1 and 1 μg/mL (Figure 3A). No statistically significant differences (*p* > 0.05) were observed in the binding ratio between P1801 and nivolumab. The long-term receptor occupancy of P1801 by activated CD3^+^ T-cells was evaluated in cynomolgus monkeys treated with a single intravenous infusion of P1801 at 1, 5, or 20 mg/kg (Figure 3B). Almost all animals exhibited 100% receptor occupancy on Day 7 after infusion, which suggested a saturated P1801 binding on CD3^+^ T-cells in these animals. The receptor occupancy of PD-1 decreased in line with the decrease in the P1801 dose, and a generally dose- and time-dependent response was observed throughout the study. Increased drug exposure caused a longer period of saturated occupancy.

The effects of P1801 activity on immune activation were evaluated in one in vitro and one ex vivo cytokine response assay. In vitro assessment revealed that IL-2 secretion by human PBMCs in response to the superantigen SEB was enhanced by P1801 in a dose-dependent manner (Figure 3C). The increase in IL-2 secretion induced by P1801 was comparable to that induced by nivolumab and significantly greater than that induced by the IgG4 control. Among monkeys treated with a single intravenous dose of P1801 at 5 or 20 mg/kg in an ex vivo assessment, PBMCs collected after the administration of P1801 showed significantly greater SEB-induced IL-2 release than that of pre-dose and the increase in IL-2 was sustained up to 56 days after dosing (Figure 3D). In contrast, P1801 had no IL-2 stimulatory effect in the absence of the superantigen SEB. The positive controls, i.e., mouse anti-human CD3 antibody and mouse anti-human CD28 antibody, induced significantly increased cytokine production in PBMCs, as indicated by increases in the levels of IL-6, IFN-γ, IL-1β, IL-4, IL-2, IL-10, and TNF, while P1801 had limited activity in inducing these cytokines when T-cells were not activated (Supplementary Figure S4).

### 3.6. PK, Immunogenicity, and Toxicokinetics of P1801 in Cynomolgus Monkeys

**Figure 4:**
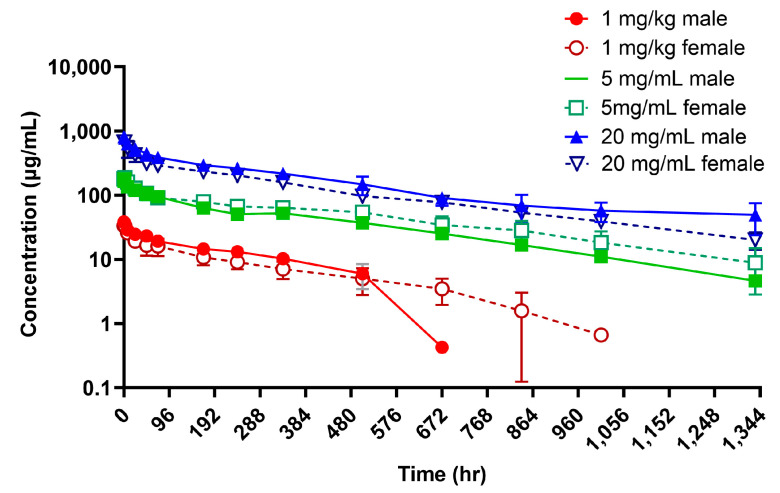
Mean serum concentrations (μg/mL) over time in cynomolgus monkeys after a single intravenous infusion of P1801 at 1, 5, or 20 mg/kg.

The PK study in cynomolgus monkeys showed a dose-dependent linear PK profile of P1801 in a single infusion at the dose from 1 mg/kg to 20 mg/kg (Figure 4). In monkeys receiving a single infusion of P1801 at 1, 5, or 20 mg/kg, the C_max_ values were 37, 186, or 746 μg/mL, respectively, and the AUC_0−last_ values were 6870, 53,000, or 178,000 h∗μg/mL, respectively (Table 1). The slope of the relationship between the dose and drug exposure was close to 1 (Supplementary Table S3). T_1/2_ ranged from 132 to 643 h. With a limited volume of distribution, approximately 0.04 to 0.06 L/kg, P1801 is thought to be mainly confined to vascular spaces. No obvious sex difference was observed in the PK profile.

Drug accumulation was observed in cynomolgus monkeys treated with a repeated dose of P1801 once weekly in the dose range from 5 to 200 mg/kg (Supplementary Table S4). Drug exposure was generally dose proportional, and a steady state was reached after four cycles of once-weekly dosing. The drug accumulation ratios at steady state ranged from 2.26 to 2.45 for AUC_0−last_ and from 1.78 to 1.98 for C_max_ when compared to the first dose. T_1/2_ ranged from 181 to 202 h in the steady state, which was generally comparable to that in the first dose. The overall frequency of induction of anti-P1801 antibody was 22.7%, without dose dependency, in a repeat-dose toxicokinetic study (Supplementary Table S5). A repeat dose of P1801 once weekly was safe and well tolerated at doses ranging from 5 to 200 mg/kg/dose. No effect on body weight or other P1801-related clinical findings was observed, except for a dose-dependent decrease in food consumption observed during the treatment period in the higher-dose groups. With no consequent changes in body weight, the observation of low food consumption was considered a non-adverse effect. No abnormalities were noted in clinical pathology or the macroscopic or microscopic examination. The no observed adverse effect level (NOAEL) of the repeat dose of P1801 once-weekly was 200 mg/kg/dose in the study.

### 3.7. Antitumor Activity of P1801 in Mice

**Figure 5:**
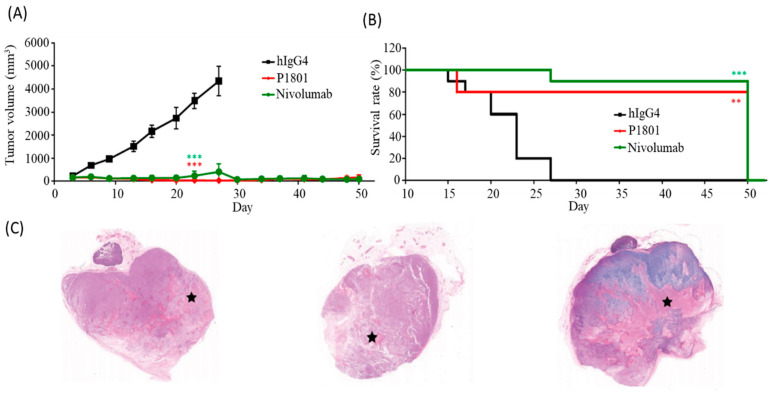
The intraperitoneal injection of P1801 or nivolumab twice a week at a dose of 12 mg/kg significantly inhibited tumor growth (**A**) and prolonged overall survival (**B**) in hPD-1 HuGEMM mice bearing HuCell MC38-hPDL1 tumor cells. Representative images of H&E staining of tumors in hIgG4, P1801, and nivolumab groups (left, middle, and right, respectively. The necrosis areas are marked with stars) were presented (**C**). ** *p* < 0.01; *** *p* < 0.001; compared to the hIgG4.

In transgenic HuGEMM mice harboring the human PD-1 gene that were subcutaneously implanted with MC38 tumor cells harboring human PD-L1, the antitumor activity of P1801 was assessed. P1801 significantly inhibited tumor growth. P1801 was administered intraperitoneally twice per week at 12 mg/kg for six doses. Compared to the isotype control, P1801 significantly inhibited tumor growth (Figure 5A). The rate of tumor growth inhibition (TGI) reached 99% on Day 23 after initial dosing, similar to that of mice treated with nivolumab at 12 mg/kg twice per week (Supplementary Table S6). According to the follow-up data, more than half of the mice treated with P1801 or nivolumab achieved a complete response (CR; 6 of 12 mice in the P1801 group and 8 of 12 mice in the nivolumab group). No tumor recurrence was observed in either the P1801 or nivolumab group at the end of observation. The P1801 group had a significantly longer median survival than the isotype control group (Figure 5B). The median survival time was 23 days in the isotype control group while it had not been reached in P1801 group at the end of the 50-day survival follow-up period. Representative tumor images are shown in Figure 5C. These data showed that P1801 has significant antitumor activity.

## 4. Discussion

PD-1 is a well-recognized immune checkpoint molecule that is expressed mainly on the surface of activated T-cells [4,5]. The binding of PD-1 to PD-L1/2 downregulates cytotoxic T-cell-mediated immunological response [6]. Tumor cells pathologically upregulate the expression of PD-L1/2 to evade T-cell-mediated immune responses. Treatment with anti-PD-1 and anti-PD-L1/2 therapeutic antibodies can restore the antitumor immunological response by interfering with the interactions between PD-1 and PD-L1/2 [6,8]. 

Cancers often derive from infection of viruses carrying viral oncogenes. An important feature of viral oncogenes during carcinogenesis is its capability of modulating the PD-1/PD-L1 interaction pathway, leading to an immune checkpoint activation that render cancer cells and viruses capable of escaping the immune responses against them. Viral oncogenes often upregulate PD-L1 and create an immunosuppressive microenvironment beneficial to tumorigenesis. Examples include the upregulation of PD-L1 by the EBV viral oncoprotein LMP1 in EBV-associated cancers such as cHLs and nasopharyngeal carcinoma (NPC) [13,40]. LMP1 was shown to up-regulate PD-L1 via the STAT3, AP-1, and NF-κB pathways in NPC cells [40]. Expression of PD-L1 was higher in EBV-positive NPC cell lines than that of the negative cell lines. In addition, mutant viral oncogene homologue KRAS was suggested to induce PD-L1 expression in lung adenocarcinoma cells. Overexpression of KRAS mutant G12V elevated PD-L1 expression in lung cancer cells [14]. Higher levels of PD-L1 were observed in both tissues harboring mutant KRAS and tumor tissue-derived CD4^+^ and CD8^+^ T-cells in mouse models. Mechanically, KRAS mutation can lead to the upregulation of PD-L1 via the modulation of the progression of epithelial-to-mesenchymal transition (EMT) through p-ERK signaling. Patients with NSCLC showed prolonged OS after the first-line treatment of the anti-PD-1 antibody pembrolizumab [15]. Furthermore, patients carrying the KRAS G12C mutation had improved overall response rate and longer progression-free survival (PFS) and OS compared to patients with mild-type KRAS. Due to the promising antitumor activity, the anti-PD-1 approach is considered as one of the most effective treatments for late-stage cancers, including cHLs, NSCLC, and HCC [9,10,11]. 

P1801 is a novel recombinant anti-human PD-1 IgG4 humanized monoclonal antibody that specifically binds to PD-1. IgG is predominantly used in the current anti-cancer antibody-based therapeutics due to its strong ability to trigger NK cell–mediated ADCC and macrophage-mediated antibody-dependent phagocytosis [41]. Human IgG has four subclasses, i.e., IgG1, IgG2, IgG3, and IgG4, which differ primarily in their constant regions, especially in the hinge and CH2 domains. IgG1 exhibits the highest affinity for all Fcγ receptors, followed by IgG3, IgG2, and IgG4. IgG1 is highly effective in activating ADCC and antibody-dependent cellular phagocytosis (ADCP) [41]. In contrast, IgG4 shows strong affinity only for FcγRI, with significantly weaker affinities for the other receptors, making it a poor inducer of Fc-mediated effector functions. The IgG4 subclass is therefore the preferred antibody backbone for the anti-PD-1 antibodies due to its weak ability in triggering ADCC and CDC in the case of off-target binding to other tissues. Notably, all approved anti-PD-1 antibodies used in cancer therapy are IgG4-based, including cemiplimab, nivolumab, and pembrolizumab [10,11,12]. We therefore chose human IgG4 as the backbone of P1801.

P1801 inhibits the interaction of human PD-1 with PD-L1/2 with minor ADCC and no CDC, which could prevent the potential toxicity caused by the ADCC- or CDC-mediated lysis of normal immune cells that express PD-1 [42]. This observation implies that P1801 may show different characteristics regarding triggering the severe immune-related side effects observed with existing anti-PD 1 antibodies in clinical settings. It is worthwhile to note that the receptor occupancy and IL-2 secretion by active T-cells, which measure the T-cell immunity, showed similar intensities between P1801 and the marketed anti-PD-1 antibody nivolumab. Notably, P1801 did not induce the significant release of inflammatory cytokines from unactive PBMCs, which further suggested that P1801 may not cause severe cytokine release syndrome resulting from inflammatory cytokine release [43]. The specific binding of P1801 to lymphocytes, but not to other cell types, observed in the tissue cross-reactivity assay further ruled out the potential toxicity caused by nonspecific interactions. In the epitope binding study, the partial blocking activity of P1801 for the binding of benchmark antibodies, pembrolizumab, and nivolumab to PD-1 suggests that P1801 contains a unique binding site in PD-1. We are now studying the structure of the P1801 and PD-1 binding complex to confirm the finding. It is tempting to speculate that the binding of P1801 to a distinct epitope in PD-1 can result in a unique biological profile. Therefore, we are also interested in further studying whether the distinct binding profile of P1801 can contribute to a greater antitumor response or a lower level of drug resistance compared to the commercially used antibodies. Future preclinical and clinical studies will be required to further explore the differences between P1801 and currently approved PD-1 antibodies.

The drug exposure of P1801 was generally proportional to the dose. A steady state was reached after four cycles of a once-weekly dose of P1801, and the drug accumulation ratio was approximately two. T_1/2_ ranged from 124 to 210 h. Overall, the PK profile of P1801 is comparable to that of nivolumab [38]. A repeat-dose toxicity study showed the good safety and tolerability of P1801 in both rats and cynomolgus monkeys. The NOAEL was 200 mg/kg/dose in cynomolgus monkeys receiving P1801 once weekly. Considering a safety factor of 6, the maximum permitted starting dose in a human clinical trial will be 10.75 mg/kg [44]. These data support the clinical development of P1801. 

The blockade of the PD-1 signaling pathway has been shown to be clinically effective as a monotherapy. The approved indications include melanoma, cHLs, NSCLC, and HCC [9,10,11]. In addition, clinical evidence has indicated that the combined use, with either chemotherapy, e.g., platinum-based chemotherapy, or targeted therapy, such as axitinib or lenvatinib, has greater efficacy than anti-PD-1 antibody alone [9,10,11]. The use of dual immune checkpoint therapies, i.e., nivolumab in combination with ipilimumab, is also approved for the treatment of melanoma or NSCLC [9]. Combining a KRAS inhibitor, such as sotorasib, with an anti-PD-1 agent revealed promising efficacy in NSCLC patients with mutant KRAS [45]. KRAS inhibitors may enhance the expression of PD-L1 and make the cancer cells more susceptible to immune checkpoint inhibition. The combination of a therapeutic anti-PD-1 antibody with an IFN-based therapy is another promising treatment regimen for solid tumors. Type 1 IFNs, including IFN-α and IFN-β, also have antiviral and immunity-based antitumor activities. They can stimulate immunity-based antitumor activities by enhancing antigen presentation and activating cytotoxic CD8^+^ T-cells and NK cells [46,47,48]. The TME is highly immunosuppressive [49]. IFNs can modulate the TME by upregulating the secretion of the chemokine CCL4 from tumor cells or inducing a high-glucose microenvironment that promotes the transcription of T-cell costimulatory molecules [50,51]. This modulation could help recruit cytotoxic CD8^+^ T-cells to infiltrate the TME, facilitating the antitumor effect of an anti-PD-1 therapy. The immune-relevant effects together with antiviral activities support the combination use of IFN-based therapies with anti-PD-1 antibodies for the treatment of viral oncogene-induced tumors and other types of cancer. In addition, type 1 IFN signaling, as shown with IFN-β, can selectively inhibit the cell cycle S-phase progression in most types of tumor cells by activating an intra-S-phase checkpoint accompanied by senescence entry [3,52,53]. Furthermore, type 1 IFN signaling can activate apoptotic pathways to induce tumor cell apoptosis as shown by the adenoviral vector-mediated IFN-β overexpression [54]. Therefore, the IFN-induced direct antitumor activities can also add to the therapeutic effect during IFN combinations with anti-PD-1 antibodies in cancer treatment. 

In a previous Phase 1 clinical study, we assessed a sequential combination therapy with ropeginterferon alfa-2b (ropeg), a novel polyethylene glycol-conjugated recombinant proline-IFN-α [55,56], and nivolumab in patients with hepatitis B-related HCC who had undergone surgical tumor removal [57]. The sequential treatment of ropeg and nivolumab was well tolerated and showed clinical feasibility for the combination. Therefore, the sequential combination therapy with our new anti-PD-1 antibody P1801 and ropeg is a reasonable next step for developing a treatment regimen for patients with solid tumors. We are initiating a new Phase I/II study to evaluate the safety and clinical activity of the sequential combination therapy consisting of ropeg and P1801 in patients with advanced solid tumors.

## 5. Conclusions

P1801 is a novel, recombinant anti-human PD-1 IgG4 monoclonal antibody that specifically inhibits the interaction of human PD-1 with PD-L1 and PD-L2. It has a minor effect on the induction of ADCC and CDC. P1801 was well-tolerated at doses up to 200 mg/kg/dose in cynomolgus monkeys. It had a significant antitumor effect in mice bearing PD-1 humanized colorectal tumors. Our study provides support for P1801 clinical development, and we are initiating a Phase I/II clinical study of P1801 with ropeg for the treatment of patients with advanced cancer.

## Figures and Tables

**Table 1:** Single-dose (first dose) pharmacokinetic parameters of P1801 in cynomolgus monkeys.

PK Parameters (First Dose)	1 mg/kg n = 4	5 mg/kg n = 4	20 mg/kg n = 4
C_max_ (μg/mL)			
Mean	37.0	186.0	746.0
SD	3.11	35.5	89.9
T_max_ (h)			
Mean	1.00	1.00	0.50
SD	1.15	1.15	1.00
T_1/2_ (h)			
Mean	132.0	282.0	643.0
SD	14.1	46.7	334.0
CL (mL/min/kg)			
Mean	0.00248	0.00155	0.00168
SD	0.000675	0.000308	0.000520
Vss (L/kg)			
Mean	0.0398	0.0415	0.0631
SD	0.00965	0.00513	0.00783
AUC_0−last_ (h∗μg/mL)			
Mean	6870	53,000	178,000
SD	1310	11,100	41,600
AUC_inf_ (h∗μg/mL)			
Mean	7010	55,900	218,000
SD	1650	13,400	81,700

## Data Availability

The data presented in this study are available in the text and the Supplementary Materials.

## Supplementary Materials

The following supporting information can be downloaded at: https://www.mdpi.com/article/10.3390/cancers16173052/s1, Figure S1: Kinetics analysis and competition of purified monoclonal antibody binding to PD-1; Figure S2: The representative image for the detection of target cells expressing human PD-1 in flow cytometry examination of the ADCC study; Figure S3: The representative image for the detection of target cells expressing human PD-1 in flow cytometry examination of the CDC study; Figure S4: In vitro assessment of cytokine induction by P1801 in non-activated human PBMC; Table S1: In vitro binding affinity assessment of P1801 to human PD-1, FcRn and C1q; Table S2: Tissue cross-reactivity of P1801 towards human and cynomolgus monkey tissues; Table S3: Dose proportionality for P1801 drug exposure in cynomolgus monkeys treated with a single dose of P1801; Table S4: Single-dose (first dose) and multiple-dose (last dose) pharmacokinetic parameters in a four-week repeat toxicokinetic study in cynomolgus monkeys; Table S5: The induction of anti-P1801 antibody in cynomolgus monkeys treated with repeated doses of P1801; Table S6: Tumor size in hPD-1 HuGEMM/HuCell MC38-hPDL mice treated with P1801 or nivolumab.
