# Supplementary material for: A Novel Monoclonal Antibody against PD-1 for the Treatment of Viral Oncogene-Induced Tumors or Other Cancer
Source: Cancers (Basel). 2024 Sep 1;16(17):3052. doi: 10.3390/cancers16173052 (PMC11393876; doi:10.3390/cancers16173052)
Supplement: Supplementary file 1 [file cancers-16-03052-s001.zip › cancers-3164780-supplementary.pdf]

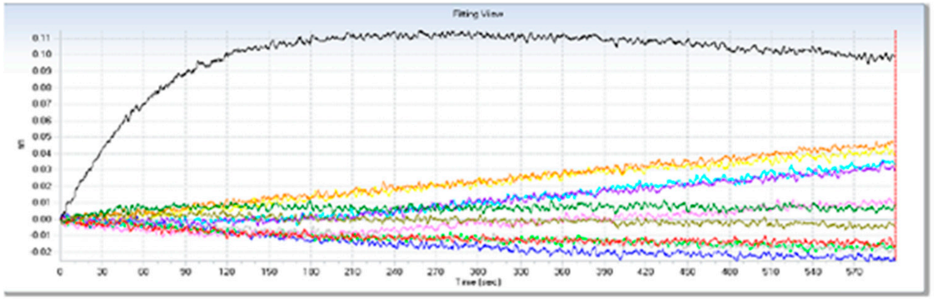

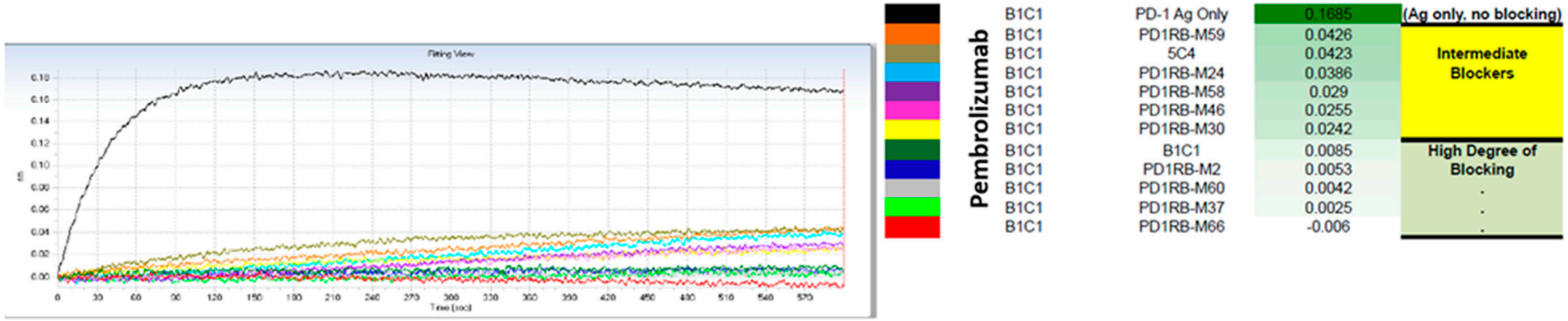

	Benchmark	Anti-PD-1		
	Sensor Loaded Ab	Blocking Ab	Ranked Response (nm)	Comment
Nivolumab	5C4	PD-1 Ag Only	0.0985	(Ag only, no blocking)
	5C4	PD1RB-M59	0.0453	Intermediate Blockers
	5C4	PD1RB-M30	0.0415	
	5C4	PD1RB-M24	0.0339	
	5C4	PD1RB-M58	0.0314	
	5C4	PD1RB-M46	0.0105	
	5C4	B1C1	0.0063	
	5C4	5C4	-0.0042	High Degree of Blocking
	5C4	PD1RB-M66	-0.0146	
	5C4	PD1RB-M37	-0.0159	.
	5C4	PD1RB-M60	-0.0177	.
	5C4	PD1RB-M2	-0.0239	.
Pembrolizumab	B1C1	PD-1 Ag Only	0.1685	(Ag only, no blocking)
	B1C1	PD1RB-M59	0.0426	Intermediate Blockers
	B1C1	5C4	0.0423	
	B1C1	PD1RB-M24	0.0386	
	B1C1	PD1RB-M58	0.029	
	B1C1	PD1RB-M46	0.0255	
	B1C1	PD1RB-M30	0.0242	
	B1C1	B1C1	0.0085	High Degree of Blocking
	B1C1	PD1RB-M2	0.0053	
	B1C1	PD1RB-M60	0.0042	.
	B1C1	PD1RB-M37	0.0025	.
	B1C1	PD1RB-M66	-0.006	.

Supplemental Figure S1. Kinetics Analysis and competition of Purified Monoclonal Antibody Binding to PD-1. In the biosensor system, alterations in the number of bound molecules induce a real-time shift in the interference pattern. When 5C4 was loaded onto sensors, 5C4, PD1RB-M2, -M37, -M60, and -M66 demonstrated the most substantial epitope blocking. Similarly, when sensors were loaded with B1C1, the notable blocking activity was observed, with B1C1, PD1RB-M2, -M37, -M60, and -M66. Notably, regardless of the benchmark antibody used for loading, PD1RB-M59 displayed the least amount of epitope overlap, indicating a unique and distinct binding profile, albeit with partial blocking activity.

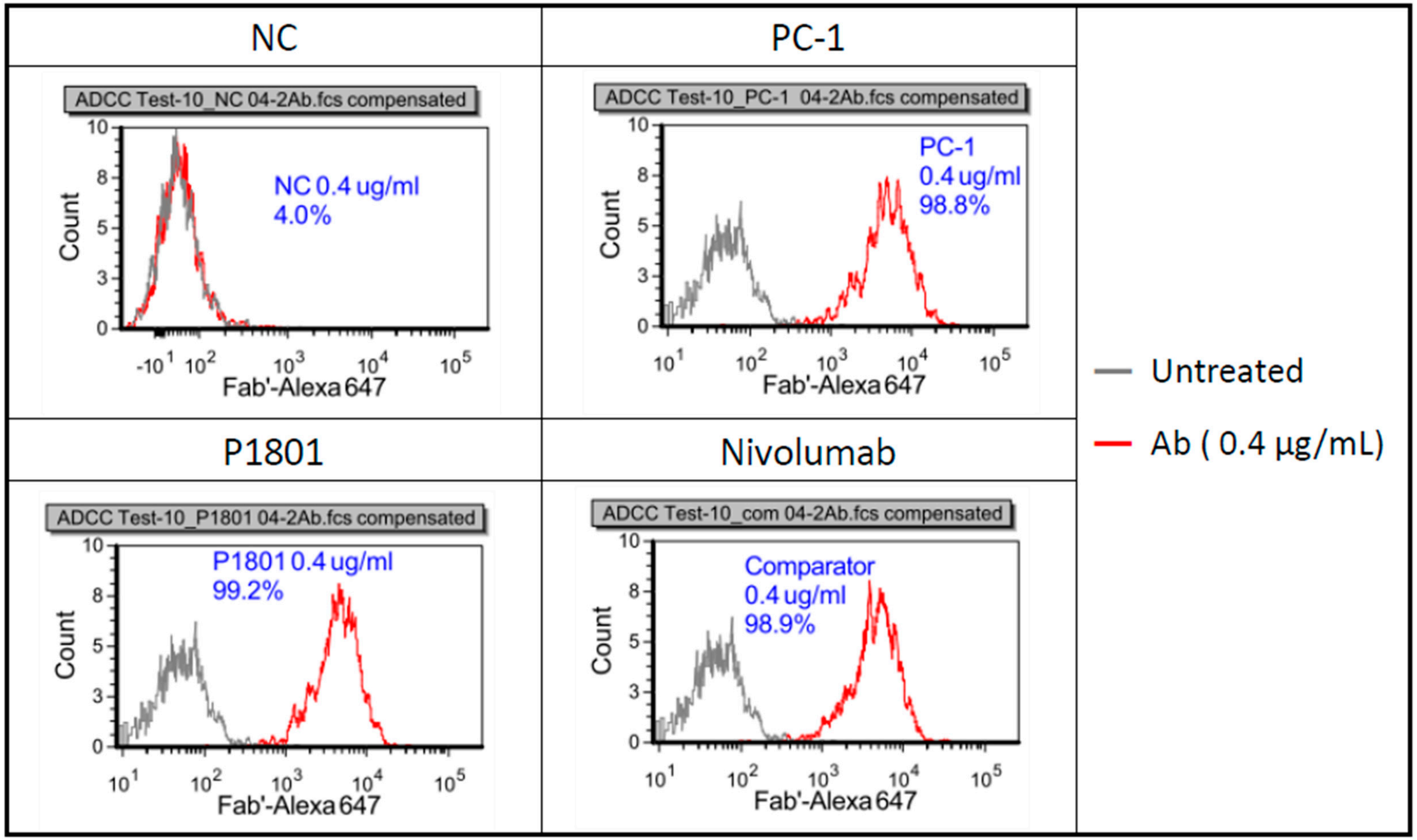

Supplemental Figure S2. The representative image for the detection of target cells expressing human PD-1 in flow cytometry examination of the ADCC study.

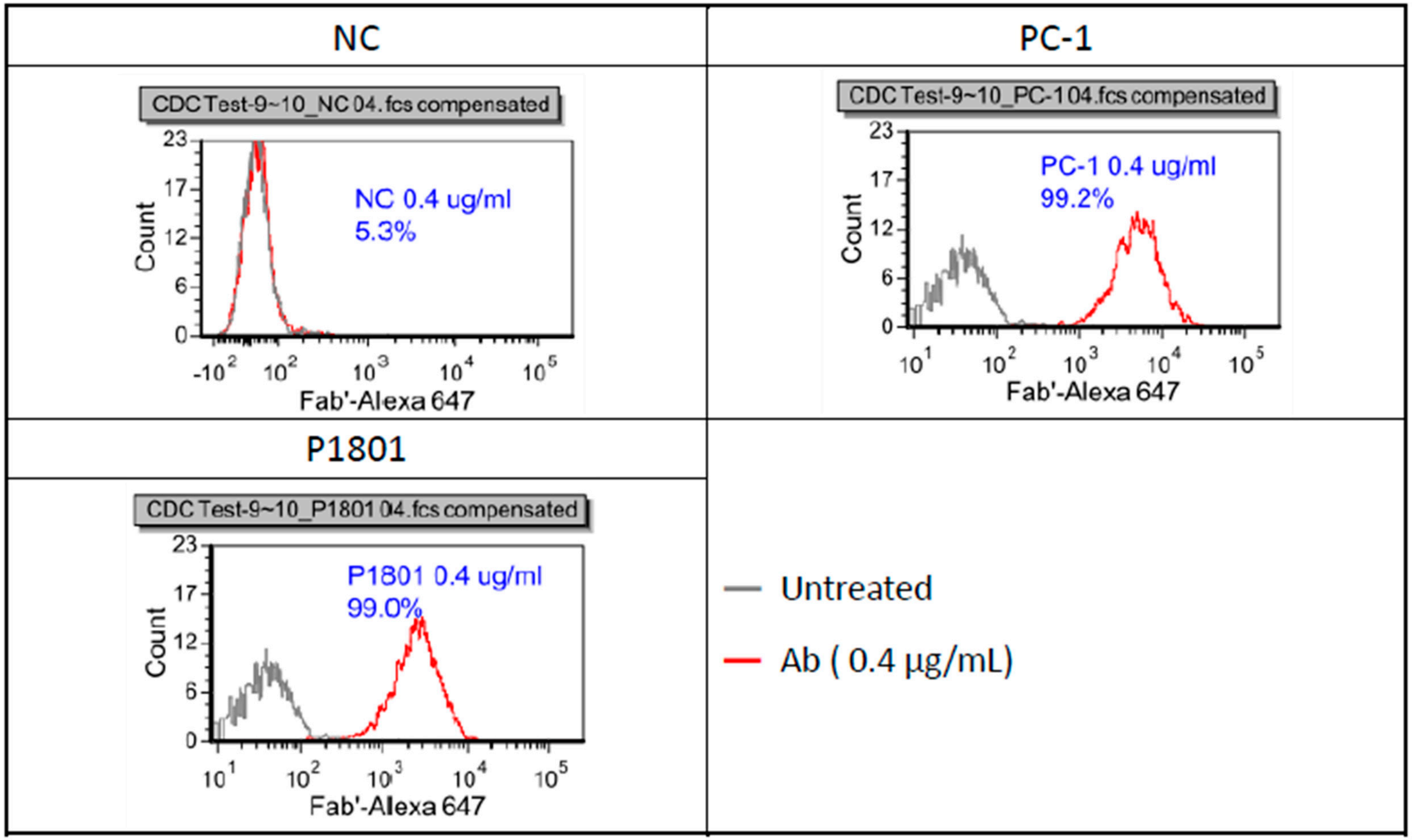

Supplemental Figure S3. The representative image for the detection of target cells expressing human PD-1 in flow cytometry examination of the CDC study.

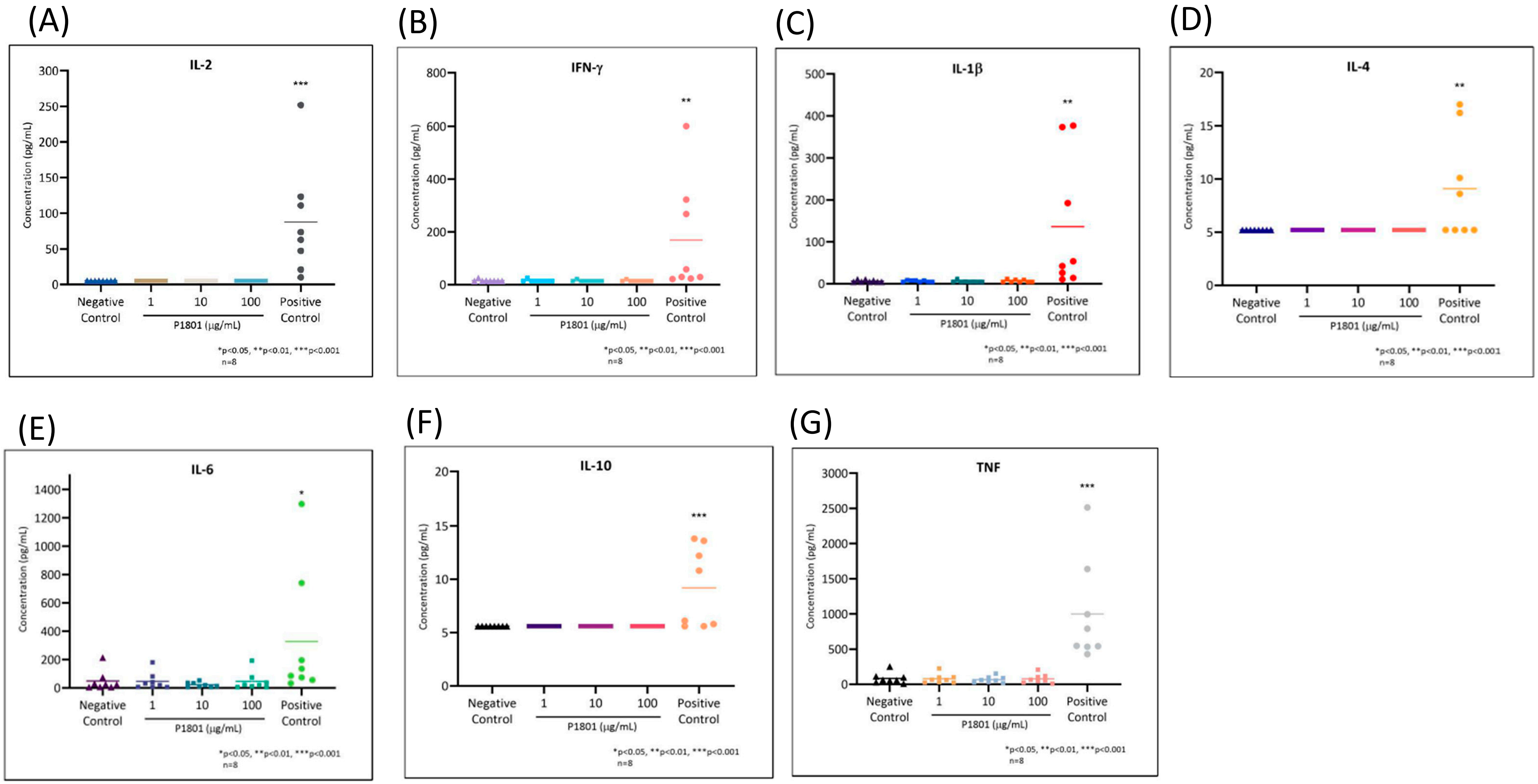

Supplemental Figure S4. In vitro assessment of cytokine induction by P1801 in non-activated human PBMC. Human PBMCs were co-incubated with P1801 for 24 hours at 1, 10 or 100 μg/mL. The cytokines including IL-2 (A), IFN-γ (B), IL-1β (C), IL-4 (D), IL-6 (E), IL-10 (F), and TNF (G) released in the supernatant were measured.

Supplementary Table S1. In vitro binding affinity assessment of P1801 to human PD1, FcRn and C1q.

Ligand	Analyte	Chi^2 (RU^2)	Ka (1/ms)	Kd (1/s)	KD (M)	R_{max} (RU)
P1801	Human PD-1	1.66E-01	5.04E+05	1.26E-03	2.50E-09	47.3
FcRn	P1801	3.54E-01	2.43E+05	6.16E-02	2.53E-07	38.1
C1q	P1801	6.09E-01	1.00E+06	5.68E-02	5.68E-08	33.7

Supplemental Table S2. Tissue cross-reactivity of P1801 towards human and cynomolgus monkey tissues.

Tissues with specific positive, membranous staining* with P1801 on lymphocytes	
Human **	Cynomolgus Monkeys **
Adrenal, urinary bladder, blood cells, bone marrow, breast, brain-cerebellum, fallopian tube, oesophagus, gastric antrum, gastric body, duodenum, ileum, colon, kidney, liver, lung, lymph node, ovary, parathyroid, parotid, pituitary, placenta, prostate, skin, spleen, testis, thymus, tonsil, ureter & uterus (cervix & endometrium)	Oesophagus, gastric antrum, gastric body, duodenum, ileum, colon, lung, lymph node, parotid, pituitary, spleen, thymus, thyroid, tonsil & uterus (cervix & endometrium)

* In a proportion of the human and cynomolgus monkey tissues, nonspecific staining was occasionally observed with P1801-Biotin, IgG4-Biotin and the antibody diluent in scattered mononuclear cells/histiocytes; the presence of which was not considered to impact the assessment of specific positive staining in lymphocytes.

**The positive staining of lymphocytes, observed in both species, was predominantly in lymphoid tissues and/or tissues with associated lymphoid structures.

Supplemental Table S3. Dose proportionality for P1801 drug exposure in cynomolgus monkeys treated with a single dose of P1801.

Drug exposure parameter	Dose Level (mg/kg)			Dose Proportionality
	1	5	20	
C_{max} (μg/mL)	37	186	746	1.00:5.03:20.20 (fold)
$AUC_{0\text{-}last}$ (hr*μg/mL)	6870	53000	178000	1.00:7.71:25.90 (fold)

Supplemental Table S4. Single-dose (first dose) and multiple-dose (last dose) pharmacokinetic parameters in a four-week repeat toxicokinetic study in cynomolgus monkeys.

**PK Parameters (first dose)**	**5 mg/kg n=6**	**50 mg/kg n=6**	**200 mg/kg n=6**
C_{max} (µg/mL)			
Mean	119	1180	4210
T_{max} (hr)			
Mean	1	1	1
$T_{1/2}$ (hr)			
Mean	175	228	226
$AUC_{0\text{-}las}$ (hr*µg/mL)			
Mean	10700	102000	390000
**PK Parameters (steady state)**	**5 mg/kg n=6**	**50 mg/kg n=6**	**200 mg/kg n=6**
C_{max} (ng/mL)			
Mean	206	2210	8210
T_{max} (hr)			
Mean	1	1	1
$T_{1/2}$ (hr)			
Mean	202	193	181
$AUC_{0\text{-}last}$ (hr*µg/mL)			
Mean	23700	253000	902000

Supplemental Table S5. The induction of anti-P1801 antibody in cynomolgus monkeys treated with repeated doses of P1801.

ADA frequency	5 mg/kg<br>N=6	50 mg/kg<br>N=6	200 mg/kg<br>N=10	Total<br>N=22
n (%)	2 (33.3)	3 (50)	0 (0)	5 (22.7)

Supplemental Table S6. Tumour size in hPD-1 HuGEMM/HuCell MC38-hPDL mice treated with P1801 or nivolumab.

Treatment	Tumour size (mm^3, mean±SEM) on Day 23	Tumour growth inhibitor (%)*	P value**
Isotype control (hIgG4) 12 mg/kg; i.p.; BIW (N=10)	3489.56 ± 333.14	-	-
P1801, 12 mg/kg, i.p., BIW (N=10)	39.39 ± 26.80	99	<0.001
Nivolumab, 12 mg/kg; i.p.; BIW (N=10)	236.69 ± 199.59	93	<0.001

* Tumour growth inhibition was calculated by the formula below.
(Mean tumour size of hIgG4 −mean tumour size of P1801 or nivolumab)/mean tumour size of hIgG4
** p value was calculated to compare the group means between study treatment, i.e., P1801 or nivolumab, and the isotype control group. The Bartlett test was used to check the homogeneity of variance and normality. If the p-value in the Bartlett test was no less than 0.05, ANOVA and a two-sample t-test were used to compare group means. If the p-value in the Bartlett test was less than 0.05, the Kruskal-Wallis test and Wilcoxon rank sum test were used to compare group means.
